# Database Mining Detected a Cuproptosis-Related Prognostic Signature and a Related Regulatory Axis in Breast Cancer

**DOI:** 10.1155/2022/9004830

**Published:** 2022-10-19

**Authors:** Baohong Jiang, Hongbo Zhu, Wenjie Feng, Zhixing Wan, Xiaowen Qi, Rongfang He, Liming Xie, Yuehua Li

**Affiliations:** ^1^Department of Pharmacy, The First Affiliated Hospital, Hengyang Medical School, University of South China, Hengyang, Hunan, China; ^2^Department of Medical Oncology, The First Affiliated Hospital, Hengyang Medical School, University of South China, Hengyang, Hunan, China; ^3^Department of Pathology, The First Affiliated Hospital, Hengyang Medical School, University of South China, Hengyang, Hunan, China

## Abstract

**Background:**

Breast cancer is the frequent cause of disease burden related to cancer among women. It affects one in 20 women globally and up to one in eight women in high-income countries. Cuproptosis is a copper-induced modality of mitochondrial cell death that is involved in tumor proliferation and metastasis.

**Methods:**

To construct a prognostic cuproptosis-related signature, LASSO Cox regression analysis was employed. Additionally, ceRNA was developed with an aim of exploring the possible lncRNA-miRNA-mRNA regulatory axis in breast cancer.

**Results:**

The expression of FDX1, DLD, DLAT, LIAS, LIPT1, GLS MTF1, and PDHA1 was downregulated, while CDKN2A expression level was elevated in breast cancer in contrast with normal tissue. We furthermore reviewed the genetic mutation landscape of genes linked to cuproptosis in breast cancer. Prognosis analysis revealed poor OS and RFS rates in breast cancer patients with elevated levels of CDKN2A and PDHA1 and low levels of MTF1, DLD, LIPT1, and FDX1. We then constructed a cuproptosis-related signature with six genes (DKN2A, MTF1, PDHA1, DLD, LIPT1, and FDX1) for breast cancer, which predicted the OS rate with an accuracy that ranged from medium to high. Further analysis demonstrated a significant correlation between the cuproptosis-related prognostic signature and pTNM stage, MSI score, drug sensitivity, TMB score, and immune cell infiltration. Moreover, we identified the lncRNA XIST/miR-92b-3p/MTF1 regulatory axis for breast cancer.

**Conclusion:**

Multiomics approaches were used to create a cuproptosis-related signature with six genes (DKN2A, MTF1, PDHA1, DLD, LIPT1, and FDX1) for breast cancer. We discovered the lncRNA XIST/miR-92b-3p/MTF1 regulatory axis for breast cancer, which has not yet been investigated previously.

## 1. Introduction

Breast cancer is the predominant cause of disease burden related to cancer among women. It affects one in 20 women globally and up to one in eight women in high-income countries [1]. With the introduction of surgical treatments, radiotherapy, chemotherapy, and targeted and endocrine therapy for breast cancer, the overall death rate from breast cancer has decreased significantly [2–5]. However, the incidence of breast cancer has increased in recent years [6]. Early diagnosis and timely treatment are essential for improvements in the prognosis of breast cancer. An increasing number of studies have identified novel effective biomarkers for the diagnosis of breast cancer; these biomarkers include miRNAs, lncRNAs, and circulating tumor DNA [7–9]. Advanced progress has been made in diagnostic approaches and therapeutic strategies. However, the outcome of breast cancer patients with metastatic disease is still poor, as it accounts for over 90% of breast cancer-related deaths [10]. Therefore, it is important to mine prognostic biomarkers and the potential mechanisms of invasive breast cancer.

Cuproptosis is a copper-activated modality of mitochondrial cell death that was first reported by Tsvetkov et al. in 2022 [11]. The accumulation of copper induces mitochondrial lipoylated protein aggregation as well as Fe-S cluster protein destabilization, causing a distinctive type of cell death [12]. Increasing evidence has revealed that higher levels of copper in many malignancies versus normal tissues lead to the modulation of cancer cell proliferation, growth, and metastasis [13]. Some scholars have even suggested copper as a vulnerable point that could serve as a target for arresting cancer development [14]. Limited studies on the significance of cuproptosis-related genes in the progression as well as prognosis of breast cancer have been conducted in accordance with our best knowledge.

In recent years, database mining using the TCGA and GEO databases has been a promising strategy to clarify human cancer-related molecular mechanisms and relevant prognostic markers [15–18]. This study utilized multiple biological methods to figure out the prognostic value of cuproptosis-related genes as well as the relevant regulatory axis in breast cancer.

## 2. Materials and Methods

### 2.1. Dataset and Preprocessing

According to a study by Tsvetkov et al., we obtained 10 cuproptosis-related genes (DLD, DLAT, FDX1, GLS, LIAS, LIPT1, MTF1, CDKN2A, PDHA1, and PDHB). The RNA-seq data of breast invasive carcinoma (BRCA) were downloaded from the TCGA (https://cancergenome.nih.gov/) and the International Cancer Genome Consortium (ICGC; https://www.icgc-argo.org/) databases. Those cases who had already received chemoradiotherapy were excluded from our study. This was followed by the normalization of gene expression profiles to transcripts per kilobase million (TPM) values. The mRNA levels of cuproptosis-related genes in BRCA versus normal tissues were measured by Student's *t*-test. The visualization of the findings was accomplished by R (version 4.0.3) with the ggplot2 package.

### 2.2. Genetic Mutations and GO and KEGG Pathway Analyses

The TCGA database provided the SNV and CNV data of BRCA. In order to evaluate the genetic mutations of cuproptosis-related genes in BRCA, the “maftools” package in the R software played a central role. In addition, to identify the potential functions of cuproptosis-related genes, we conducted GO and KEGG analyses.

### 2.3. Cuproptosis-Related Prognostic Gene Signature Development

Overall survival (OS) and recurrence-free survival (RFS) analyses were conducted to evaluate the prognostic value of cuproptosis-related genes in BRCA with the log-rank test computing hazard ratios (HRs), the *p* values, and 95% CIs. After identifying potential prognostic biomarkers, we did the LASSO Cox regression analysis based on these genes to create a ferroptosis-related prognostic gene signature. Utilizing the median value as the cutoff value, we categorized BRCA cases into two groups on the basis of the median value as the cutoff value, which was computed as the sum of coefficients × cuproptosis-related gene expression. The OS curve was drawn by the Kaplan-Meier method, and the area under the curve (AUC) was analyzed with time ROC analysis. We also verified the cuproptosis-related prognostic gene signature using the ICGC dataset.

### 2.4. Hub Gene Analysis

Pearson's correlation test was conducted to establish the link between cuproptosis-related prognostic genes and the proportion of immune cells in the TIMER (https://cistrome.shinyapps.io/timer/) database. Moreover, it determined the link between cuproptosis-related prognostic genes and the IC_50_ of small molecules in the Cancer Therapeutics Response Portal (CTRP) database. The Spearman correlation test in this experiment evaluated the link between cuproptosis-related prognostic genes and tumor mutational burden (TMB)/microsatellite instability (MSI) in TCGA.

### 2.5. Potential Regulatory Axis Analysis

Four miRNA target prediction databases, miRDB (http://mirdb.org/), TargetScan (https://www.targetscan.org/), starBase (http://starbase.sysu.edu.cn/), and miRWalk (http://mirwalk.umm.uni-heidelberg.de/), were utilized to explore the miRNA targets of the hub gene MTF1. Moreover, two lncRBA databases, starBase (http://starbase.sysu.edu.cn/) and the LncBase module of the DIANA tool (http://carolina.imis.athena-innovation.gr/), were utilized to explore the lncRNA targets that interact with miRNAs. We additionally evaluated the expression as well as the prognostic value of miRNAs and lncRNAs using Student's *t*-tests and the Kaplan-Meier method utilizing the TCGA BRCA dataset.

### 2.6. Verification Analyses

Considering MTF1 expression and other clinical characters, univariate and multivariate analyses were conducted to identify prognostic factors. We also verified the prognostic value of MTF1 in the OS and RFS analyses using GSE20685 dataset. Moreover, the protein level of MTF1 expression was determined with The Human Protein Atlas (https://www.proteinatlas.org/). The immunohistochemistry of MTF1 in normal and cancer tissue was obtained in the “tissue” and “pathology” modules of The Human Protein Atlas.

### 2.7. Statistical Analyses

The mRNA levels of cuproptosis-related genes in BRCA versus normal tissues were subjected to analysis using Student's *t*-test. The Kaplan-Meier method was applied to draw the survival curve with the log-rank test computing the *p* values, hazard ratio (HR), and 95% CI. LASSO Cox regression analysis was done to create a cuproptosis-related prognostic gene signature. Pearson's correlation test ascertained the link between gene expression and immune cell infiltration and drug sensitivity. The Spearman correlation test also determined the link between gene expression and TMB/MSI score.

## 3. Results

### 3.1. The Expression and Mutation Landscape of Cuproptosis-Related Genes in Breast Cancer

**Figure 1:**
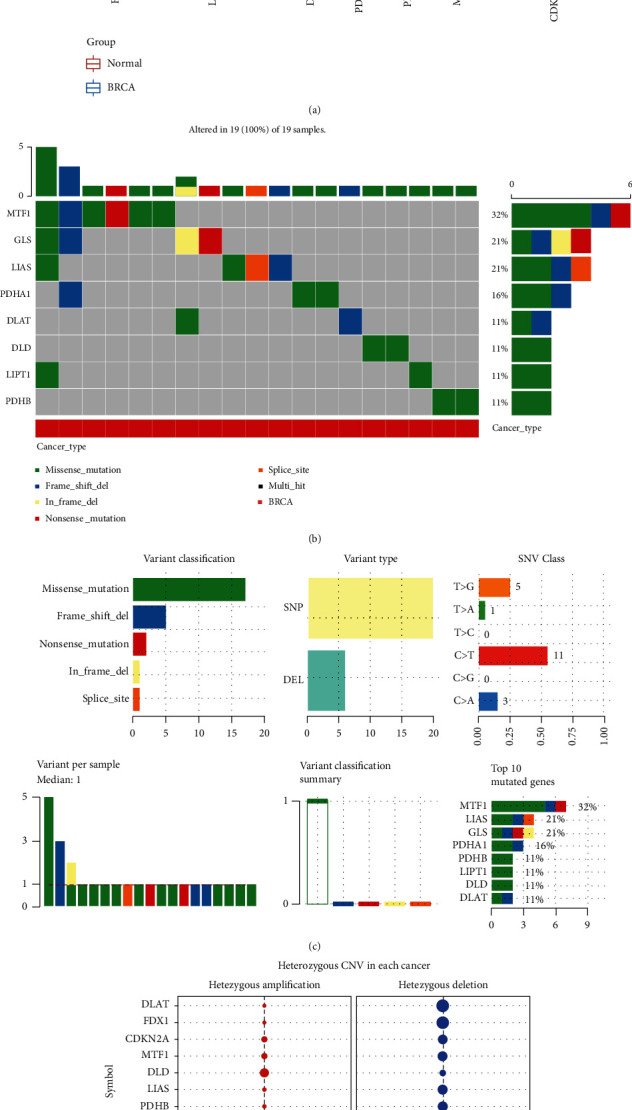
The expression and mutation landscape of cuproptosis-related genes in breast cancer. (a) The mRNA level of the cuproptosis-related gene in breast cancer versus normal tissues. (b, c) SNV investigation of the cuproptosis-related gene in breast cancer. (c) CNV investigation of the cuproptosis-related gene in breast cancer. ^∗^*p* < 0.05; ^∗∗^*p* < 0.01; ^∗∗∗^*p* < 0.001; ^−^*p* > 0.05.

The expression landscape is demonstrated in Figure 1(a). The expression level of 9 of 10 cuproptosis-related genes was altered in breast cancer tissues (all *p* < 0.0001). The data indicated that the expression of PDHA1, FDX1, GLS, DLD, DLAT, LIAS, LIPT1, and MTF1 was downregulated in breast cancer versus normal tissue. CDKN2A expression level was elevated in the cancerous breast tissues in contrast to normal tissues. Mutation analysis showed that 32% of breast cancer samples had MTF1 genetic mutations. The next most commonly mutated genes were GLS (21%), LIAS (21%), and PDHA1 (16%) (Figure 1(b)). Further analysis depicted that missense mutations and C>T mutations were the predominant variant classification and SNV class, respectively (Figure 1(c)).  Figure 1(d) shows the results of the CNV analysis. These results indicate that most of the 10 cuproptosis-related genes had homozygous amplifications, and the DLD gene had a widespread homozygous deletion.

### 3.2. GO and KEGG Analyses

**Figure 2:**
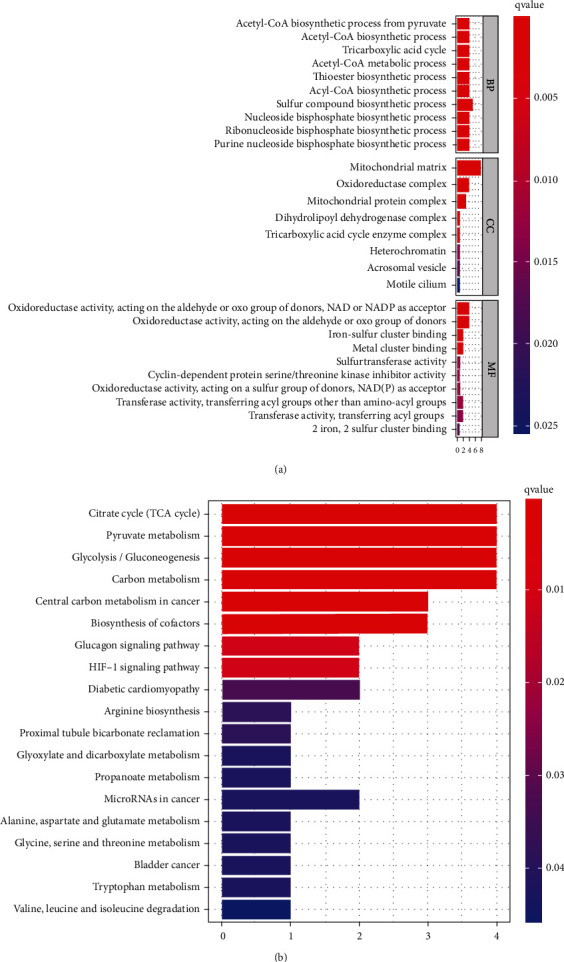
The enriched items in GO and KEGG pathways analyses. (a) The enriched items in GO analysis. (b) The enriched items in KEGG analysis. BP: biological process; MF: molecular function; CC: cellular component.

To further elucidate the possible roles of cuproptosis-related genes, functional enrichment analyses, including GO and KEGG pathway analyses, were conducted. As a result, GO analysis demonstrated the participation of these cuproptosis-related genes in the acetyl-CoA biosynthetic process from pyruvate, acetyl-CoA metabolic process, tricarboxylic acid cycle, mitochondrial matrix, oxidoreductase complex, oxidoreductase activity, and metal cluster binding (Figure 2(a)). Furthermore, KEGG pathway analysis revealed the involvement of the TCA cycle, pyruvate metabolism, gluconeogenesis, carbon metabolism, HIF-1 signaling pathway, miRNAs in cancer, and glucagon signaling pathway (Figure 2(b)).

### 3.3. Development of a Cuproptosis-Related Prognostic Signature in Breast Cancer

**Figure 3:**
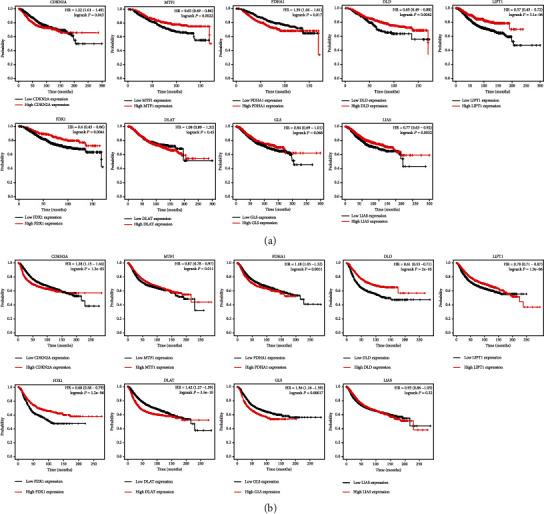
The prognostic value of the cuproptosis-related genes in breast cancer. (a) The result of OS analysis of cuproptosis-related genes in breast cancer. (b) The result of recurrence-free survival evaluation of cuproptosis-related genes in breast cancer.

**Figure 4:**
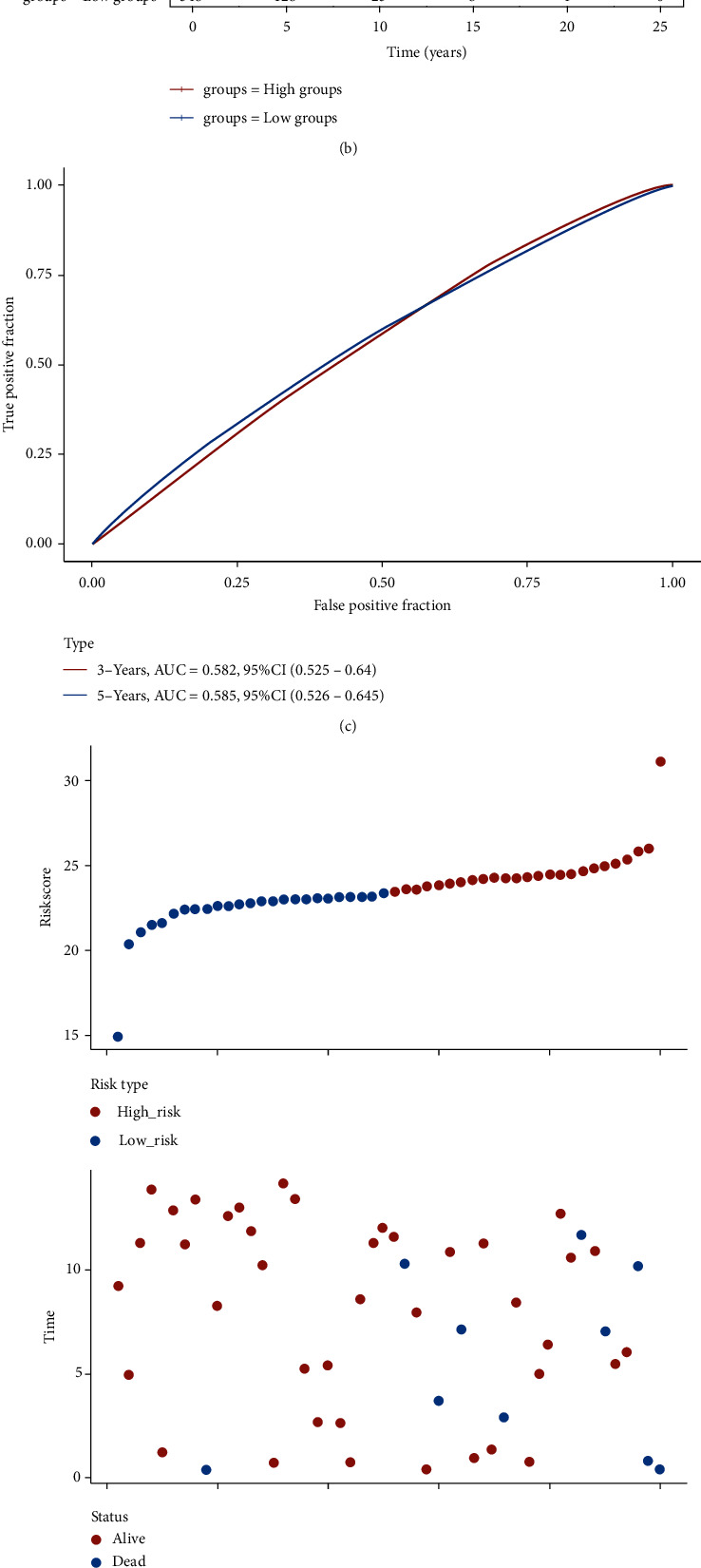
Cuproptosis-related prognostic signature in breast cancer. (a) The patient survival status, risk score distribution, and cuproptosis-related gene expression profile of prognostic signature in TCGA cohort. (b, c) The breast cancer patients' survival curve with high/low risk score and ROC curve of prognostic signature in TCGA cohort. (d) The patient survival status, risk score distribution, and cuproptosis-related gene expression profile of prognostic signature in ICGC cohort. (e, f) The breast cancer patients' survival curve with high/low risk score and ROC curve of prognostic signature in ICGC cohort.

OS analysis and RFS were utilized to evaluate the prognostic value of differentially expressed cuproptosis-related genes in BRCA.  Figure 3(a) shows the findings of the OS analysis. These results reveal a poor OS rate in breast cancer patients with elevated levels of CDKN2A (*p* = 0.043, HR = 1.22) and PDHA1 (*p* = 0.0042, HR = 1.39). However, breast cancer patients with elevated levels of MTF1 (*p* = 0.0022, HR = 0.65), DLD (*p* = 0.0042, HR = 0.65), LIPT1 (*p* = 3.1*e*^−6^, HR = 0.37), FDX1 (*p* = 0.0041, HR = 0.6), and LIAS (*p* = 0.0052, HR = 0.77) had a better OS rate (Figure 3(a)).  Figure 3(b) shows the results of the RFS analysis. These results reveal a poor RFS rate in breast cancer patients with high levels of CDKN2A (*p* = 1.3*e*^−5^, HR = 1.28), PDHA1 (*p* = 0.0051, HR = 1.18), and GLS (*p* = 0.00017, HR = 1.36). However, patients with breast cancer who had high levels of MTF1 (*p* = 0.011, HR = 0.87), DLD (*p* = 2*e* − ^10^, HR = 0.61), LIPT1 (*p* = 1.9*e*^−6^, HR = 0.78), and FDX1 (*p* = 1.2*e*^−6^, HR = 0.68) had a better RFS rate (Figure 3(b)). This evidence suggests that CDKN2A, MTF1, PDHA1, DLD, LIPT1, and FDX1 are potential biomarkers for breast cancer. Utilizing these possible prognostic biomarkers, we executed a LASSO Cox regression analysis, and afterward, a cuproptosis-related prognostic signature containing these 5 prognostic biomarkers was constructed for breast cancer. The risk score of every TCGA patient was determined utilizing the formula: risk score = (0.1001)∗FDX1 + (−0.3109)∗LIPT1 + (0.1966)∗DLD + (0.1678)∗PDHA1 + (0.076)∗MTF1 + (−0.0802)∗CDKN2A. All TCGA BRCA samples were classified into high- and low-risk groups.  Figure 4(a) outlines the risk scores, gene expression, and survival status of the prognostic signature. As expected, BRCA patients in the TCGA cohort with a greater risk score were affirmed to have a poor OS rate (Figure 4(b), *p* = 0.000148, HR = 1.895), with AUCs of 0.582 and 0.582 in the 3-year and 5-year ROC curves, correspondingly (Figure 4(c)). To validate further this prognostic signature, we adopted a verification analysis using the ICGC dataset. The risk score of every TCGA patient was determined with the formula: risk score = (−0.1729)∗FDX1 + (−0.4454)∗LIPT1 + (2.9672)∗DLD + (0.3425)∗PDHA1 + (−0.8096)∗MTF1 + (−0.1194)∗CDKN2A. The risk score of the ICGC sample, gene expression of the verification prognostic signature, and survival status are displayed in Figure 4(d). As anticipated, BRCA patients in the ICGC cohort with an elevated risk score demonstrated a poor OS rate (Figure 4(e), *p* = 0.0165, HR = 13.055), with AUCs of 0.728 and 0.701 in the 3-year and 5-year ROC curves, correspondingly (Figure 4(f)).

### 3.4. Hub Gene Analysis

**Figure 5:**
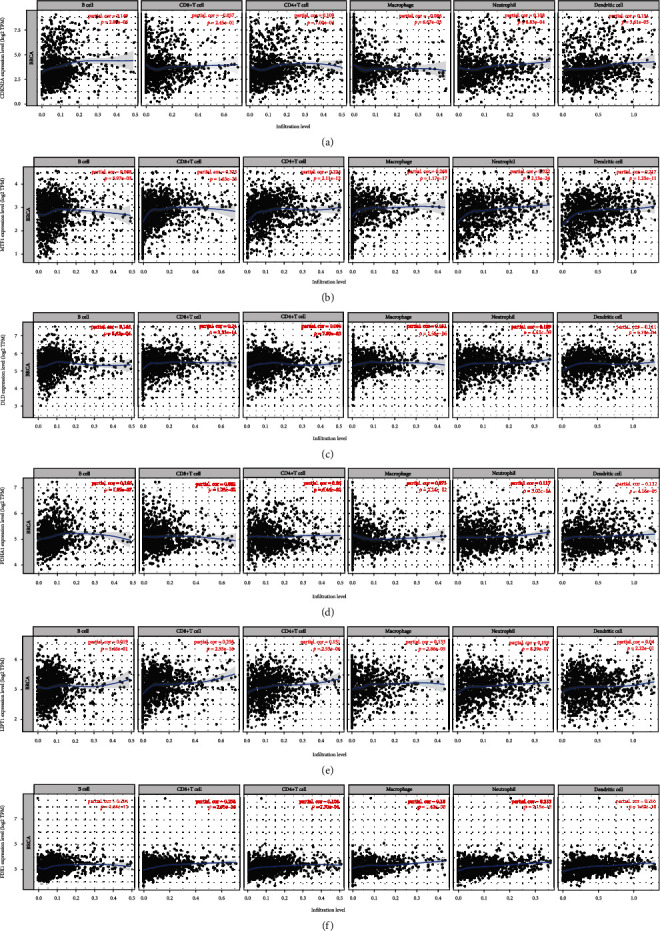
Immune cell infiltration of cuproptosis-related prognostic signature in breast cancer. Link between the expression of CDKN2A (a), MTF1 (b), PDHA1 (c), DLD (d), LIPT1 (e), and FDX1 (f) and the abundance of immune cells in breast cancer.

**Figure 6:**
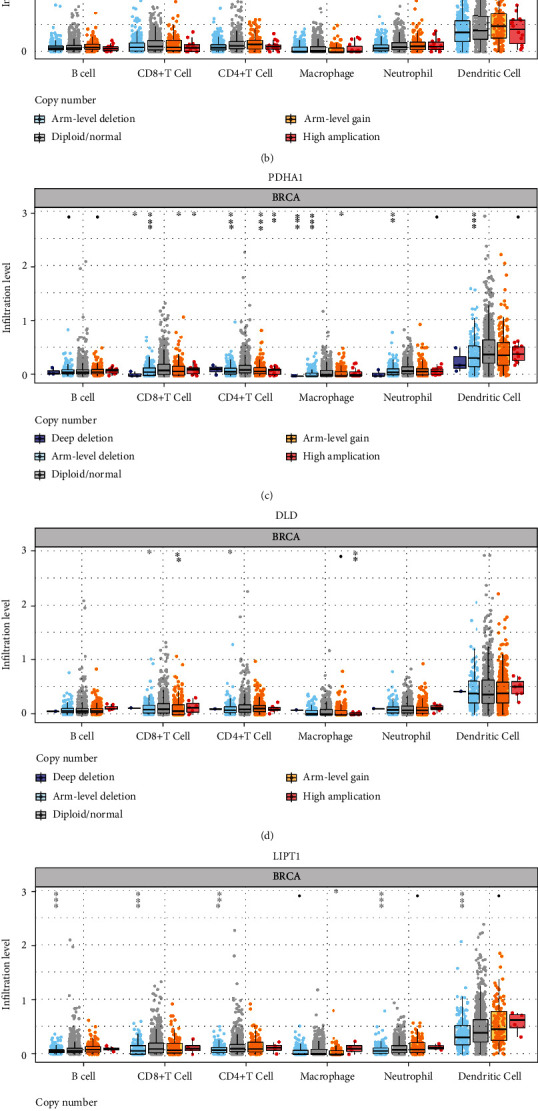
Immune cell infiltration analysis of cuproptosis-related prognostic signature in breast cancer. Correlation between CNV of CDKN2A (a), MTF1 (b), PDHA1 (c), DLD (d), LIPT1 (e), and FDX1 (f) and immune cell infiltration in breast cancer.

**Figure 7:**
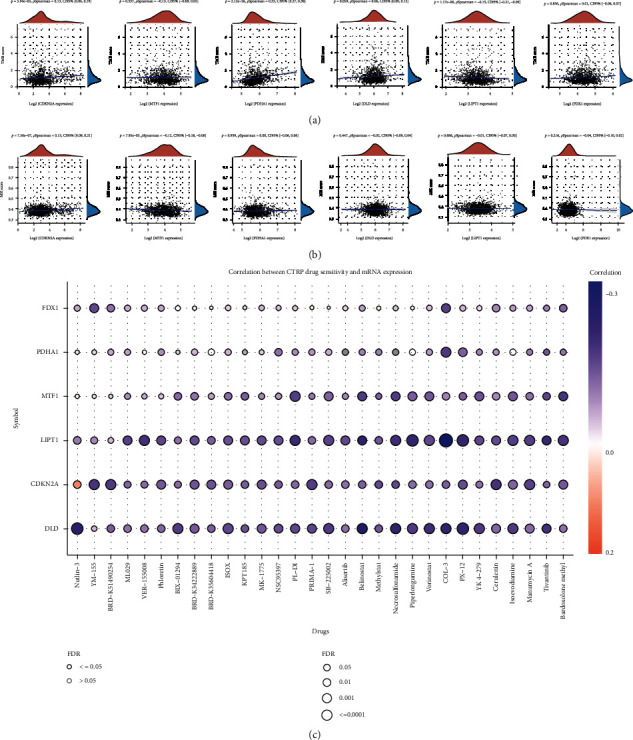
TMB, MSI, and drug sensitivity analyses of cuproptosis-related prognostic signature in breast cancer. (a) Link between the expression of cuproptosis-related prognostic signature and TMB score in breast cancer. (b) Link between the expression of cuproptosis-related prognostic signature and MSI score in breast cancer. (c) Link between the expression of cuproptosis-related prognostic signature and drug sensitivity in breast cancer. TMB: tumor mutation burden; MSI: microsatellite instability.

**Figure 8:**
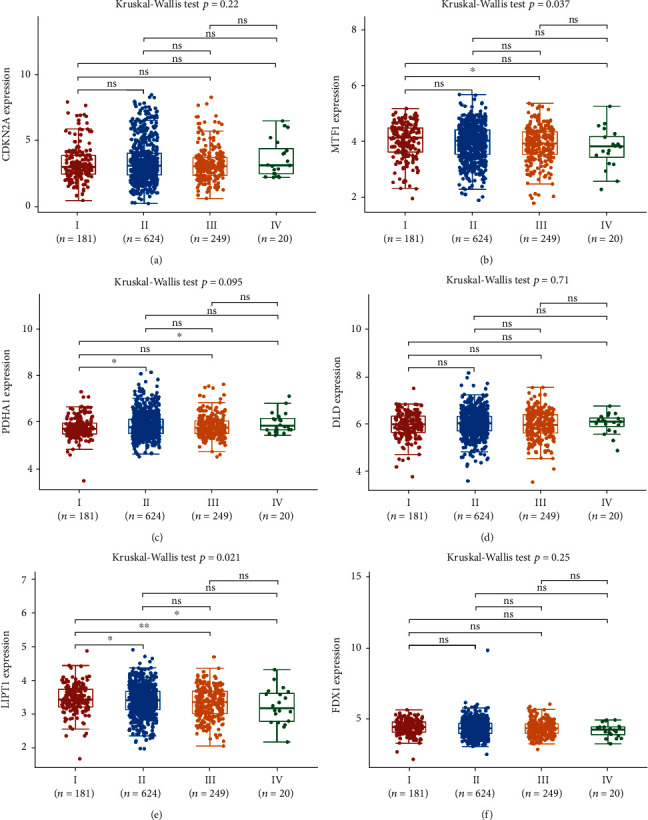
The expression of cuproptosis-related prognostic signature in various groups of patients with breast cancer. The expression of CDKN2A (a), MTF1 (b), PDHA1 (c), DLD (d), LIPT1 (e), and FDX1 (f) in breast cancer patients in different pTNM stages.

We afterward analyzed the link between the cuproptosis-related prognostic signature and immune infiltration. We ascertained that CDKN2A expression was positively correlated with the abundance of B cells, CD4^+^ T cells, dendritic cells, and neutrophils (Figure 5(a)). The abundance of B cells, CD8^+^ T cells, CD4^+^ T cells, macrophages, dendritic cells, and neutrophils was elevated as the expression of MTF1 (Figure 5(b)), DLD (Figure 5(c)), and FDX1 (Figure 5(f)) increased. Moreover, the data suggested a positive link between PDHA1 expression and the proportion of B cells, CD8^+^ T cells, macrophages, dendritic cells, and neutrophils (Figure 5(d)). A positive link was established between LIPT1 expression and immune cell infiltration (CD8^+^ T cells, CD4^+^ T cells, macrophages, and neutrophils) (Figure 5(e)). Interestingly, somatic cell copy number alterations in the cuproptosis-related prognostic signature inhibited the infiltration level of some immune cells (Figures 6(a)–6(f)). Increasing evidence suggests that TMB and MSI are predictive markers for determining the efficacy of tumor immunotherapy in cancer [19, 20]. TMB analysis revealed a remarkable link between TMB score and the expression of CDKN2A, PDHA1, and LIPT1 (Figure 7(a), all *p* < 0.05). The data affirmed a remarkable correlation between the MSI score and the expression of CDKN2A and MTF1 (Figure 7(b), all *p* < 0.05). To ascertain the potential of the cuproptosis-related prognostic signature as a drug scanning target for breast cancer, we additionally carried out a drug sensitivity analysis. This analysis implied that the low expression of DLD, CDKN2A, LIPT1, and MTF1 was positively linked to drug resistance to CTRP (Figure 7(c)). To clarify the potential role of the cuproptosis-related prognostic signature in the development of breast cancer, we evaluated their link with the pTNM stage. The results revealed that the expression of MTF1 (Figure 8(b), *p* = 0.037) and LIPT1 (Figure 8(e), *p* = 0.021) decreased as the pTNM stage increased in breast cancer. No remarkable correlation between the pTNM stage and the expression of CDKN2A (Figure 8(a)), PDHA1 (Figure 8(c)), DLD (Figure 8(d)), or FDX1 (Figure 8(f)) was established. These data suggest that MTF1 and LIPT1 may participate in breast cancer progression.

### 3.5. Verification of the Expression and Prognostic Value of MTF1 in Breast Cancer

**Figure 9:**
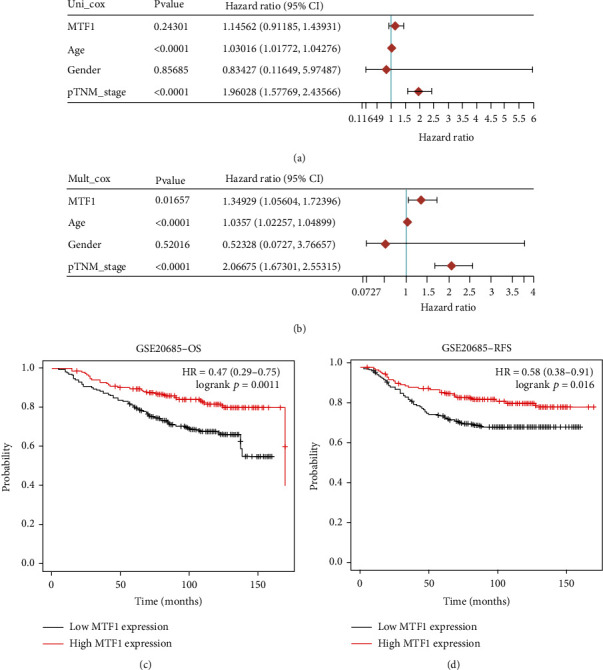
Verification of the prognostic value of MTF1 in breast cancer. (a, b) Univariate analysis and multivariate analysis taking into account clinical characters and MTF1 expression. (c, d) The OS and RFS in patients with breast cancer patients with MTF1 expression levels that are high and low.

The aforementioned data suggested that MTF1 and LIPT1 may participate in breast cancer progression. We selected MTF1 for further analysis. We afterward validated the prognostic value of MTF1 in breast cancer. Univariate as well as multivariate analyses indicated that MTF1 expression, age, and pTNM stage were independent factors influencing the breast cancer patient's prognoses (Figures 9(a) and 9(b)). Consistent with the previous data, immunohistochemistry revealed that MTF1 was medium staining in normal tissue while it was low staining in breast cancer tissue (Supplementary Figure 1). In prognosis analysis of GSE20865 cohort, the data affirmed a better OS and RFS rate in breast cancer patients with elevated MTF1 expression levels (Figures 9(c) and 9(d), *p* < 0.05).

### 3.6. lncRNA-miRNA-mRNA Regulatory Axis Analysis

**Figure 10:**
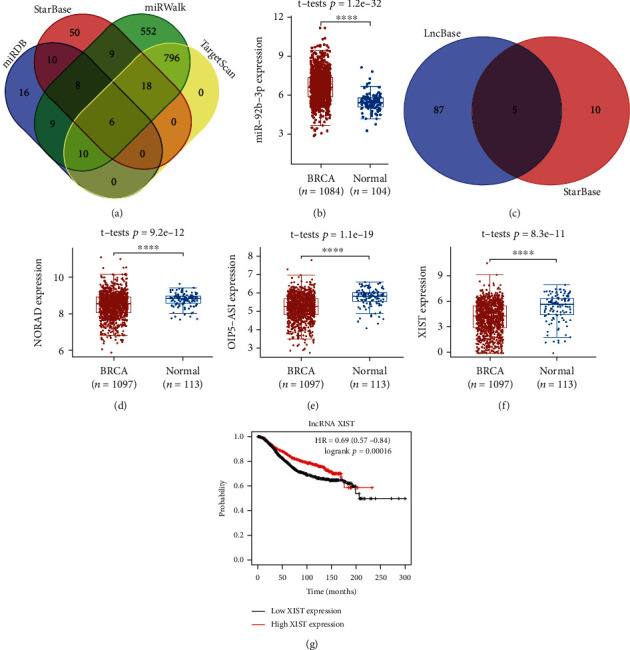
lncRNA-miRNA-mRNA regulatory axis in breast cancer. (a) The miRNA targets anticipated by TargetScan, miRWalk, and starBase. (b) The miR-92b-3p expression in breast cancer. (c) LncBase and starBase predicted lncRNA targets. (d–f) The expression of NORAD, OIP5-AS1, and XIST in breast cancer. (g) The prognostic value of lncRNA XIST in breast cancer.

Finally, we conducted an lncRNA-miRNA-mRNA regulatory axis analysis to elucidate the role of MTF1 in breast cancer. Based on the predicted miRNA targets of the starBase, miRWalk, TargetScan, and miRDB databases, six miRNAs (miR-92b-3p, miR-302a-3p, miR-25-3p, miR-367-3p, miR-520a-3p, and miR-4319) were suggested as the miRNA targets of MTF1 (Figure 10(a)). Among these miRNAs, only miR-92b-3p was differentially expressed in breast cancer, so we selected miR-92b-3p as a miRNA target of MTF1 (Figure 10(b)). We additionally evaluated the lncRNA targets of miR-92b-3p, and the findings suggested that the lncRNAs DAPK1-IT1, AC005394.2, XIST, NORAD, and OIP5-AS1 are potential lncRNA targets of miR-92b-3p (Figure 10(c)). Among these five lncRNAs, the XIST (Figure 10(d), *p* = 9.2*e*^−12^), NORAD (Figure 10(e), *p* = 1.1*e*^−9^), and OIP5-AS1 lncRNAs (Figure 10(f), *p* = 8.3*e*^−11^) were expressed differentially in breast cancer. Moreover, patients with breast cancer and with elevated XIST levels demonstrated a better survival (Figure 10(g), *p* = 0.00016). Thus, the XIST lncRNA was the most likely potential lncRNA target of miR-92b-3p. The lncRNA XIST/miR-92b-3p/MTF1 regulatory axis may be important in breast cancer progression.

## 4. Discussion

Breast cancer is the predominant malignant-related cause of disease burden among women. It affects one in 20 women globally and up to one in eight women in high-income countries [1]. The prognosis of patients diagnosed with breast cancer and metastatic disease is poor, as it accounts for over 90% of breast cancer-related deaths [10]. The mechanisms of tumorigenesis, as well as the progression of breast cancer, are still not fully understood. Therefore, it is important to mine prognostic biomarkers and potential mechanisms of invasive breast cancer. Cuproptosis is a copper-triggered modality of mitochondrial cell death that was first reported by Tsvetkov et al. in 2022 [11]. Increasing evidence has affirmed the importance of cuproptosis in the modulation of tumor cell proliferation, growth, and metastasis [13], and limited studies on the importance of cuproptosis-related genes in not only the progression but also the prognosis of breast cancer have been conducted. This study systematically studied the roles exerted by cuproptosis-related genes in breast cancer.

The gene expression profile revealed that the expression of FDX1, GLS, LIAS, LIPT1, MTF1, DLD, DLAT, and PDHA1 was downregulated, whereas the CDKN2A expression was upregulated in breast cancer in contrast with the normal tissue. Further, functional analysis demonstrated that these cuproptosis-related genes were associated with the TCA cycle, gluconeogenesis, and the HIF-1 signaling pathway in GO and KEGG analyses. In fact, studies have clarified the involvement of these genes in the advancement of cancer of the breast and its therapy. Liu et al. affirmed that HIF-1-regulated expression of calreticulin could accelerate tumorigenesis and progression in breast cancer [21]. Another study affirmed that the HIF-1 pathway participated in the regulation of cancer metabolism and survival stress [22]. The TCA cycle could affect tumor burden and invasion, thus leading to poor prognosis and a lack of targeted therapies in breast cancer [23].

Prognosis analysis revealed poor OS and RFS rates in breast cancer patients with elevated levels of CDKN2A and PDHA1 and low levels of MTF1, DLD, LIPT1, and FDX1. These findings suggest that CDKN2A, MTF1, PDHA1, DLD, LIPT1, and FDX1 are potential biomarkers for breast cancer. In fact, these cuproptosis-related genes have been found to be biomarkers in other types of cancers. In ovarian cancer, MTF1 might be a new biomarker for prompt diagnosis and aid in targeted therapy [24]. Another study suggested that CDKN2A is not only a prognostic marker but is also involved in immune infiltration in hepatocellular carcinoma [25]. In gastric cancer, a low level of PDHA1 was associated with a poor prognosis [26].

Another vital discovery of our research was that we developed a cuproptosis-related prognostic signature containing six genes (CDKN2A, MTF1, PDHA1, DLD, LIPT1, and FDX1) for breast cancer, which predicted the OS rate with an accuracy that ranged from medium to high. This is the first pyroptosis-related prognostic signature identified in human carcinoma, although numerous prognostic signatures have been discovered for breast cancer as per our best knowledge. Previous studies have identified an autophagy-related lncRNA signature that can predict prognosis in breast cancer [27]. Wang et al. also developed a prognostic signature consisting of nine ferroptosis-related genes for breast cancer [28]. Another study also identified and verified a necroptosis-related gene signature as well as an associated regulatory axis in breast cancer [29].

Our study also ascertained the lncRNA XIST/miR-92b-3p/MTF1 regulatory axis for the progression of breast cancer. The XIST lncRNA could inhibit cancer proliferation and ETM and promote apoptosis in breast cancer, as affirmed by a previous study [30]. XIST was also suggested as a likely cancer immune marker in breast cancer patients with high PD-L1 levels [31]. Moreover, miR-92b-3p can function as not only a diagnostic marker but also as a prognostic marker in breast cancer and correlates with clinical staging and tumor differentiation in breast cancer [32]. Mediated by XIST, miR-92b-3p could accelerate tumor progression in hepatocellular carcinoma [33]. Our study determined the lncRNA XIST/miR-92b-3p/MTF1 regulatory axis for breast cancer, which has not been studied before.

## 5. Conclusion

In conclusion, multiomics approaches were conducted to develop a cuproptosis-related signature with six genes (DKN2A, MTF1, PDHA1, DLD, LIPT1, and FDX1) for breast cancer. We identified the lncRNA XIST/miR-92b-3p/MTF1 regulatory axis for breast cancer, which has not been studied before.

## Data Availability

The analyzed data sets generated during the study are available from the corresponding author on reasonable request.

## Supplementary Materials

Supplementary Materials Supplementary Figure 1. The immunohistochemistry of MTF1 in breast cancer tissue and normal tissue.
