# Supplementary figures and images for: Database Mining Detected a Cuproptosis-Related Prognostic Signature and a Related Regulatory Axis in Breast Cancer
Source: Dis Markers. 2022 Oct 19;2022:9004830. doi: 10.1155/2022/9004830 (PMC9605827; doi:10.1155/2022/9004830)

Antibody HPA028689

Normal tissue

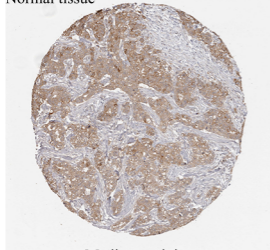

Medium staining

Breast cancer  
tissue

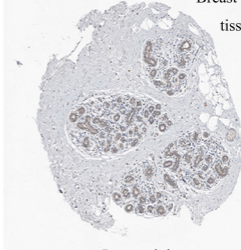

Low staining

Supplement: Supplementary Materials — Supplementary Figure 1. The immunohistochemistry of MTF1 in breast cancer tissue and normal tissue. [file 9004830.f1.pdf]
